# Masculinised Behaviour of XY Females in a Mammal with Naturally Occuring Sex Reversal

**DOI:** 10.1038/srep22881

**Published:** 2016-03-11

**Authors:** Paul A. Saunders, Thomas Franco, Camille Sottas, Tangui Maurice, Guila Ganem, Frédéric Veyrunes

**Affiliations:** 1 Institut des Sciences de l’Evolution de Montpellier, Université de Montpellier, CNRS UMR 5554, IRD, EPHE, France; 2 INSERM U1198, Université de Montpellier, Inserm, EPHE, France

## Abstract

Most sex differences in phenotype are controlled by gonadal hormones, but recent work on laboratory strain mice that present discordant chromosomal and gonadal sex showed that sex chromosome complement can have a direct influence on the establishment of sex-specific behaviours, independently from gonads. In this study, we analyse the behaviour of a rodent with naturally occurring sex reversal: the African pygmy mouse *Mus minutoides*, in which all males are XY, while females are of three types: XX, XX* or X*Y (the asterisk represents an unknown X-linked mutation preventing masculinisation of X*Y embryos). X*Y females show typical female anatomy and, interestingly, have greater breeding performances. We investigate the link between sex chromosome complement, behaviour and reproductive success in females by analysing several behavioural features that could potentially influence their fitness: female attractiveness, aggressiveness and anxiety. Despite sex chromosome complement was not found to impact male mate preferences, it does influence some aspects of both aggressiveness and anxiety: X^*^Y females are more aggressive than the XX and XX*, and show lower anxiogenic response to novelty, like males. We discuss how these behavioural differences might impact the breeding performances of females, and how the sex chromosome complement could shape the differences observed.

With two copies of the X chromosome in females versus one X and a Y chromosome in males, male heterogamety is the norm in mammals. The X and the Y are very different both in size and gene content as the result of a long differentiation from an ancient autosomal pair^1^. They also differ from autosomes in that they are enriched in genes that affect sexual differentiation, reproduction, brain development, behaviour and cognition^2^,^3^,^4^,^5^,^6^. Over the last decade, several studies have highlighted the direct influence of sex chromosome genes on the establishment of sexually dimorphic behaviours^7^, findings that contrast with the classical view that sex differences are due to the sole action of gonadal hormones during development^8^. The relative impact of sex chromosome complement versus gonadal sex on behaviour has been assessed using different transgenic laboratory mouse strains, such as the “Four Core Genotypes” model (FCG), in which a *Sry*-deleted Y chromosome and an autosomal *Sry* transgene produce XX and XY^−*Sry*^females (XXF and XYF) and XX^+ *Sry*^ and XY males (XXM and XYM)^9^. These studies reveal that while some sex differences in behaviour are influenced by gonadal sex, for instance chemo-investigation of bedding (XXM and XYM investigate more than XXF and XYF), others, such as certain aspects of aggressiveness, are influenced by sex chromosome complement (XYM and XYF are more aggressive than XXM and XXF)^10^. The study of FCG and other mouse models showed an influence of sex chromosomes on various other features: parental behaviour, sexual behaviour or social interactions^10^,^11^,^12^, but also on non-behavioural traits such as metabolism or brain function^13^,^14^. The differences are independent from gonadal hormones and result from the action of some Y-linked genes^15^ and/or the number of X chromosomes (e.g. one copy for XYM *vs*. two copies for XXM)^16^.

The influence of sex chromosomes on behaviour has been tested mostly on laboratory strain mice in which sex chromosome complement was genetically manipulated, but never in a species in which an unusual mode of sex determination was shaped by natural selection. In mammals, there are indeed a few natural exceptions to the standard XX/XY sex determination system (SDS). For example, fertile XY females are found in natural populations of several lemmings and South American grass mice species^17^, and both males and females are X0 in the Japanese spiny rat *Tokudaia osimensis*^18^ and the mole vole *Ellobius lutescens*^19^. Species with such unusual SDS are particularly relevant to further investigate the link between sex chromosome complement and behaviour.

The African pygmy mouse, *Mus minutoides*, a close relative of the house mouse, has recently been added to the short list of mammals with unusual SDS^20^. In populations from Southern up to Western Africa, XY females are found amongst standard XY males and XX females^21^. Sex reversal (here meaning discordance between chromosomal and phenotypic sex) of these XY females is not linked to a mutation of the male sex determining gene *Sry* nor any other Y-linked gene, but rather to the X chromosome. Cytogenetics revealed that two different X chromosomes, varying in size and structure, segregate in these populations: the ancestral X and a rearranged one named X^*^. The latter bears a still unknown mutation preventing masculinization of X*Y embryos. So while all males are XY, there are three types of females with different sex chromosome complements: XX, XX* and X*Y^20^; Sex determination is polygenic in this species^22^. The three types of females cannot be told apart phenotypically, they have a similar body mass and ano-genital distance, and all harbour typical ovarian structure^23^, which suggest similar levels of circulating hormones. However, their reproductive performances differ: unexpectedly, X*Y females produce significantly more offspring than the XX and XX* females despite the meiotic issues expected in heterogametic oocytes and the loss of unviable YY embryos. This advantage results from the production of bigger litters, a higher breeding probability when paired with a male and an earlier breeding onset^24^. The latter two features could relate to variation in female attractiveness, i.e. male preference for X*Y females, or other behavioural traits that could delay pair bonding with XX and XX* females.

In this study, we analyse several behavioural traits in the African pygmy mouse (female attractiveness, aggressiveness and anxiety in both sexes) in order to answer two questions: does sex chromosome complement affect behaviour independently from gonadal sex in a species with naturally occurring sex reversal and could behavioural differences account for the greater reproductive output of X*Y females.

## Methods

### Animals

The fifty pygmy mice (13 males and 37 females) used for this study were kept and raised at the breeding facility (CECEMA) of the University of Montpellier, France. The origin of the founder animals, and housing conditions in the colony were described previously^20^,^24^. For this study, at weaning, males and females were housed separately in cages: females were housed in same-sex groups of 3–4 individuals per cage and males set in individual cages (to prevent agonistic behaviours). They were provided with ad-libitum food and water, and light regime was set to 15:9 h (light:dark). Females were genotyped by PCR amplification of the Y-specific Sry gene and/or non-invasive fibroblast cell-culture established from skin biopsy^20^.

### Behavioural Tests

Experimental procedures were performed in accordance with European guidelines and with the approval of the French Ethical Committee on animal care and use (No. CEEA-LR- 12170).

All animals went through the different tests in the following order: Two-way choice test (i.e. Y maze) to test male preferences, resident-intruder test to test female aggressiveness, and light-dark box and open field to evaluate anxiety and exploration in both sexes. There was a minimum interval of a week between two tests, which were all conducted between 1300 h and 1900 h. The order of the tests was imposed by technical constraints; The pygmy mouse being a social species^25^, we believe that tests involving interactions are not more stressful than tests in which individuals are isolated in an unfamiliar empty arena. Sample sizes are given in Table 1, the pedigree of each animal was assessed and encounters between closely related animals were avoided. The average age of individuals at the beginning of the study was 264 +/−56 days old (mean +/−s.d.). As the oestrous state of females is thought to influence their behaviour towards conspecifs^26^,^27^,^28^,^29^, it was assessed before experiments involving encounters between individuals, using the “wet smear” method^30^: all females were receptive (in oestrous) for the male mate choice test and non-receptive for the female aggressiveness test.

#### male mate choice

Two-way male mate choice was performed using a Y maze as described by Smadja and Ganem^31^. Briefly, the apparatus consists of a transparent Y shaped maze, in which a male is introduced via the main branch (27 cm, ∆4.5 cm). At the end of the two other (secondary) branches (25 cm, ∆4.5 cm) are Plexiglas boxes (15 × 15 × 10 cm) with two receptive (in oestrous) “stimulus” females of two different genotypes. Male-female interactions are limited by perforated doors separating the boxes from the secondary branches. Each male was tested three times: once with each of the three types of pairs (XX vs. XX*, XX vs. X*Y and XX* vs. X*Y). Each stimulus female was used twice, once against each other genotype. The order of presentation of the stimuli was randomized and no male encountered the same female twice. A test started as soon as a male entered the main branch and lasted for 10 minutes. To assess male preference, the time spent in each tube (exploring, in contact with the perforated door, and in interaction with the female through the holes of the door) was measured.

#### female aggressiveness

The resident-intruder test was used to compare aggressiveness in the three types of females. This paradigm relies on the analysis of the aggressiveness of an individual in its territory (here a non-receptive “resident” female) towards an “intruder” (a male). Before the test, the female was isolated for at least a week, and then placed in a large (40 × 30 × 30 cm) transparent box with her own soiled bedding. 48 hours later, the male was introduced in the resident cage via a side door. Encounters lasted 10 minutes. Each male faced the three different types of females sequentially, with a minimum of seven days between two trials. The order in which males encountered the different female genotypes was randomized. The latency to first attack and the occurrence of agonistic behaviours (attacks and chases) directed by females were scored.

#### anxiety and exploratory behaviour

Two tests were used to assess anxiety-related and exploratory behaviours of males and females. The “Light-dark box” design consists of two adjacent boxes (23*16*10 cm) separated by a small opening (6*6 cm). The light compartment is brightly lit from above (900 lux) and covered with a transparent lid, and the dark compartment is covered with a black lid. Mice were placed in the light box, and experiments lasted 10 minutes. We considered the first two minutes as a habituation period, and recorded the latency before movement (i.e. the time it took mice to start moving at the beginning of the experiment) to assess anxiogenic response to novelty^32^. From minute two to minute 10, the time spent in the light compartment (classical measure of state anxiety in mice)^33^ and the distance covered in the whole device (to assess exploratory activity) were recorded. The “open field” is a round open area (∆50 cm) with high walls, virtually divided in two areas: the central zone (∆20 cm), and outer zone (∆20–50 cm). Mice were placed in the central zone, and tests lasted 10 minutes, under dim lighting conditions (~10 lux). In a similar way to the light-dark box, we recorded the time spent immobile at the beginning of the test to assess anxiogenic response to novelty, and from minute two to 10, the time spent in the central zone (a common measure of anxiety, as anxious individuals are expected to stay on the periphery of the field)^34^ and the total distance covered. For the two tests, up to four animals were tested simultaneously (in independent devices), in a pseudorandomised order. Each apparatus was cleaned between two trials with a 50:50 water:ethanol solution.

### Data Acquisition and Analyses

The Y maze and resident-intruder test were filmed and mice behaviours were recorded by one of us blindly and analysed with The Observer software (v 5.0.31, Noldus). For the light-dark box and open field tests, movements were tracked and recorded using an infra-red tracking device and video-tracking software (VideoTrack, v 3.10, Viewpoint).

Male mate preferences were assessed by pair comparisons of time spent in each secondary branch, in contact with each perforated doors and in interaction with each female through the holes, separately for each modality (XX vs. XX*, XX vs. X*Y and XX* vs. X*Y), using Wilcoxon signed rank tests. Correction for multiple testing was made using the Bonferroni procedure^35^.

For the two measures of aggressiveness, we carried out independent analyses, accounting for multiple testing by using the sequential Bonferroni correction^35^. The effect of genotype on the latency to first attack and the number of aggressive bouts were analysed using generalised linear mixed model with respectively (i) an exponential distribution (that applies when the variable is the time to the first occurrence of an event^36^) and (ii) a geometrical distribution (which provided best graphical fit to data). Male identity was added as a random variable, and female:male mass-ratio and trial number for the male (first, second or third) were added as fixed covariates. Model simplification was made using Likelihood ratio tests (LRT).

To analyse the variables measured in the light-dark box and open-field, we also performed independent analyses, accounting for multiple testing by using sequential Bonferroni correction. The effect of presence *vs.* absence of sex chromosomes X* and Y and their interaction (that allows to discriminate the four genotypes) was assessed on anxiety (time spent in the light box/central zone) and exploratory behaviour activity (distance covered) using univariate analysis of variance (ANOVA) after controlling for normality of the response variables (Shapiro-Wilk test^37^), and on anxiogenic response to novelty (latency before movement) using generalised linear models with an exponential distribution. Three covariates were used: the age of the individual at the time of the experiment, the order in which they were tested (12 groups of up to four animals, all animals were tested on the same day) and their position in the experimental device (upper/lower–left/right). Model simplifications were made using LRTs.

All statistical analyses were performed using R^38^.

## Results

### No Male Preference for a Given Female Genotype

Results are shown in Table 2, males spent the same amount of time in each side of the Y maze, in contact with the doors leading to the females, and interacting with the two females through the perforated doors, whichever set of females (XX vs. XX*, XX vs. X*Y or XX* vs. X*Y) they encountered in the maze. The nominally significant difference in interaction time when confronted to XX* and X*Y females (V = 56, p = 0.041) did not survive correction for multiple testing.

### X*Y Females are More Aggressive than the Others

**Figure 1:**
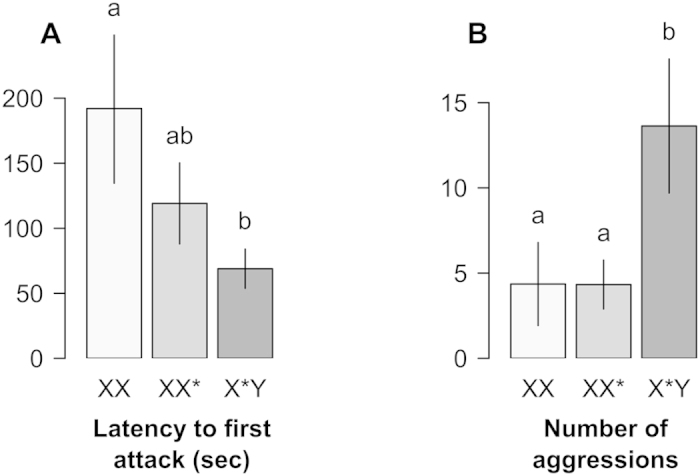
Effect of female’s genotype on Latency to first attack (A) and number of aggressions (B) by females in the resident-intruder test (mean +/− s.e.m.). The letters above the bars indicate significant differences according to Tukey’s HSD test.

Model simplifications are detailed in supplementary information 1. We found a significant effect (robust to sequential Bonferroni correction) of genotype on latency to first attack (χ^2^_2_ = 7.0, p = 0.029) and number of attacks by the resident (χ^2^_2_ = 11.00, p = 0.004) in the resident-intruder test (Fig. 1). There was no effect of any of the covariates on either trait. Tukey HSD tests were used to test post hoc differences between the three pairs of genotypes. X*Y females were significantly faster to attack males than XX females (p = 0.037) while XX* females were intermediate (XX vs. XX*: p = 0.45, XX* vs. X*Y: p = 0.27). The X*Y also attacked males more often than the XX and XX* (XX vs. X*Y, p = 0.03; XX* vs. X*Y, p = 0.01), the latter showing a similar level of aggressiveness (p = 0.93).

### Y Chromosome Effect on Behaviour in Spontaneous Exploration Tests

**Figure 2:**
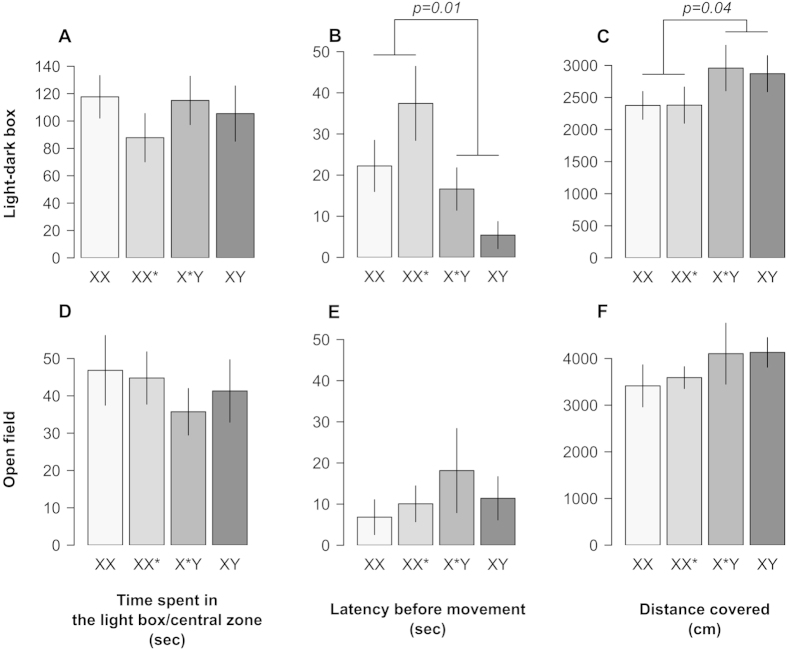
Behavioural response of mice in the Light-dark box (A–C) and Open field (D–F) paradigms (mean +/− s.e.m.). Significant differences according to the univariate ANOVAs are shown above the bars.

Variables measured in the light-dark box and open-field are presented in Fig. 2. See Fig. S1 for detailed model simplification. In the light-dark box, there was no effect of the presence vs. absence of the Y, nor of the X*, on the time spent in the light box (F_1,43_ = 0.14, p = 0.70 ; F_1,39_ = 0.11, p = 0.89). However, we found an effect of the presence of the Y chromosome on the two other variables measured: Y bearers (males and X*Y females) spent less time immobile at the beginning of the experiment (X^2^_1_ = 6.32, p = 0.01), and had an enhanced exploratory behaviour (distance covered, F_1,43_ = 4.64, p = 0.04, the effect of the Y does however not survive correction for multiple testing). There was no effect of the presence vs. absence of the X* nor of any of the covariates on these traits (S1).

Concerning the open-field, the analyses did not reveal any effect of the Y nor of the X* on any of the variables measured. Nevertheless, the non-significant trend observed for the distance covered (F_1,44_ = 2.14, p = 0.15) is similar to the differences observed in the light-dark box (Y bearers covering a greater distance). Once again, none of the covariates had an effect on the variables (S1).

## Discussion

This study addresses the influence of sex chromosome complement on female behaviour and attractiveness to males in a mammal with an unusual SDS: *Mus minutoides*. We found that X*Y females differ from XX and XX* females in respect to certain behavioural features. In fact, these sex-reversed females show an enhanced aggressiveness, and an anxiogenic response to novelty and exploratory behaviour similar to those of males, confirming the impact of sex chromosome complement on the behaviour of the African pygmy mouse.

The resident-intruder test revealed that X*Y females show a shorter attack latency than XX females, (XX* females being intermediate; Fig. 1A). They also attacked males more often than the females of the two other genotypes (Fig. 1B). In the light-dark box, sex-reversed females and males spent less time in a static posture than XX and XX* females at the beginning of the experiment (Fig. 2B). This suggests a lower anxiogenic response to novelty in individuals harbouring a Y chromosome. However, there was no effect of genotype on the anxiogenic response to novelty in the open-field. This might be explained by the differences between the two apparatus^39^, or by a form of habituation of the mice to experimental conditions. We found no effect of the Y chromosome on the other classical parameters used to assess anxiety (time spent in brightly lit compartment in the light-dark box and in the centre of the open-field; Fig. 2A,D). This underlines the complexity of anxiety related behaviours, as shown by pharmacological studies: anxiety is not an unitary phenomenon, and different aspects of anxiety rely on different neurological and hormonal pathways^40^,^41^. Finally, differences were also found in terms of exploratory behaviour in the light-dark box: X*Y females and males show greater levels of locomotor activity than the XX and XX* females (the significant effect of the Y chromosome did not survive correction for family wise type I error rate, however, the fact the same trend was observed for this trait in the two spontaneous exploration tests (Fig. 2C,F), indicates that this is likely due to a lack of power rather than a true type I error). Overall, these findings are congruent with observations showing reduced behavioural dimorphism between sex-reversed females and males in laboratory strain mice^10^,^12^,^42^.

In a previous study^24^, we showed that female genotype has an influence on breeding success: X*Y females have a higher reproductive output thanks to a greater chance of having at least one litter, an earlier breeding onset (they have their first litter in average 20 days earlier than the XX and XX*) and the production of bigger litters. Some behavioural features (e.g. attractiveness) are known to impact fitness, so we hypothesised that the differences observed in terms of probability of breeding and age at first litter might result from behavioural differences between female genotypes. Interestingly, the differences in behaviour highlighted in this paper follow the same pattern as in breeding success: X*Y differ from XX and XX* females. However, it is not straightforward how a reduced anxiety and an increased aggressiveness and exploratory behaviour might have a positive effect on fitness of X*Y females, especially as so little is known about the ecology of the African pygmy mouse^43^. The social and mating systems of this species have never been studied, which makes it hard to infer how these behavioural traits could impact breeding, but here are a few leads. Reduced anxiety of X*Y females could influence breeding success by facilitating male-female interactions. In female prairie voles, stress has been shown to inhibit pair bonding^44^, and in several other species, boldness is known to be positively correlated to fitness^45^. A greater anxiety of XX and XX* females may explain why so many of these females do not breed in our colony, and why those that do have a delayed onset of reproduction. Aggression is also related to reproduction. If female pygmy mice are territorial (many female small mammals are)^46^, the greater aggressiveness and exploratory behaviour of X*Y females could be advantageous to protect their offspring and provide adequate access to resources required for reproduction. These females could also attract more males and have more mates if their territories are bigger. Alternatively, if they live in social groups, aggressiveness could help achieving dominance and therefore a greater reproductive success. Also, in extreme cases, such as in *Mus spicilegus*, which belongs to the same genus as the African pygmy mouse, aggressiveness seems to be part of a “ritualised” sexual behaviour, triggering sexual motivation^47^. This greater aggressiveness might also be beneficial when considering the shift in sex-ratio caused by a feminizing mutation such as the X*. As some embryos with a Y become females rather than males, a female biased sex-ratio is expected in natural populations. This could alter the strength and direction of competition for mates, as mating becomes more difficult for the sex in the majority^48^. Such conditions could favour the evolution of sex-role reversal: females would benefit from being more aggressive and less anxious while competing for males and choosiness might evolve in males.

Despite male preference for X*Y females may be beneficial considering their reproductive advantage, the experiments we conducted to test male preference (Y maze) did not reveal any male preference for one type of female over another (Table 2). This does not imply that choice is absent, as our experiments were restricted to short term olfactory and visual contact, and choice can be exerted in many ways^49^. Informal observations in our laboratory colony suggest that, in contrast with laboratory mice, pair formation could take several days/weeks (e.g. it often takes several days before a male and a female can be found sharing the same nest, suggesting that it takes a certain time before they accept each other). As male preference could be a crucial feature in breeding performance (e.g. wild male house mice mated to preferred females have higher reproductive success)^50^, it should be studied more thoroughly. In addition, other behavioural experiments could be conducted, as it is not unlikely that differences in behaviour extend to other traits. For example, it has been shown using genetically manipulated laboratory mice that XY females tend to be more social than XX ones^12^,^51^. So the study of social behaviour as well as sexual and parental behaviours (which have also been found to be influenced by genes on the sex chromosomes)^10^,^52^ should help clarifying the link between sex chromosomes, behaviour and reproduction in *Mus minutoides*.

Besides the evolutionary and ecological issues raised by these results, this study also supports recent findings concerning the direct effect of sex chromosomes on behaviour. During the last decade, there has been a growing interest in the ”direct” role of the expression of sex chromosome genes on the shaping of sexual dimorphic behaviours^7^, as opposed to the “indirect” way: trough the action of gonadal hormones^53^. In the African pygmy mouse, the lack of noticeable differences between the anatomy and ovaries of XX, XX* and X*Y females^23^ could imply that all female have similar levels of circulating gonadal hormones (though this would have to be confirmed by hormonal assay). So the differences found in this study in terms of aggressiveness, anxiogenous response to novelty and exploratory behaviour is likely to result from the direct influence of genes of the Y, X and X* chromosomes on the brain.

It is notoriously hard to assign behavioural modifications to naturally occurring genetic changes^54^, but a few genes are known to have a direct effect on behaviour and would make good candidates to explain the behavioural differences found in the pygmy mouse. *Sry* and *Sts*, two genes harboured by the Y chromosome, have been shown to influence aggressiveness in mice^55^,^56^. These genes, and others of the non-recombining region of the Y, could be responsible for differences in anxiogenic response to novelty, locomotor activity and number of aggressions which dissociate Y chromosome bearers (X*Y and XY) from non-bearers (XX and XX*) in the pygmy mouse. *Sry* is a serious candidate, as it has been shown to be strongly expressed in the brain of X*Y females in the pygmy mouse^23^. In regard to attack latency, X*Y female differ from the XX, and XX* are intermediate, evoking an influence of the X*. More specifically, a gene that is expressed differently between the X and the X* could cause this pattern, as its level of expression would be intermediate in XX* females (due to random inactivation of the X). Monoamine oxidase A (*MaoA*) is an X-linked gene well known to influence behaviour: *MaoA* mice knockouts show increased aggressiveness^57^. An X-X* difference in expression of *MaoA* (or another X-linked gene) could therefore explain the differences observed in terms of attack latency. Further genetic analyses (expression of candidate genes in the brain) as well as hormonal assays (pre and post-nataly) are required to disentangle the respective implication of direct and indirect effects of genes on the breeding performance of females in this species.

## Conclusion

In this study, we show that sex chromosome complement has an impact on several behavioural traits in *Mus minutoides*, independently from gonadal sex: X*Y females show some masculinised behaviours despite their typical female anatomy.

The African pygmy mouse is a promising model to further investigate the link between behaviour and sex chromosomes, especially since unlike other animal models used for this purpose, sex-reversal is a naturally occurring phenomenon in this species. It is also the first time a behavioural study has been conducted in a mammal with an unusual SDS. Females with either XX or XY sex chromosome complement can be found in a few other mammalian species. Examining behaviour in these species, as well as extending such studies to species with other types of unusual SDS in mammals, would help to better understand the ecological and evolutionary implications of the deviation from the standard XX/XY system.

## Additional Information

**How to cite this article**: Saunders, P. A. *et al.* Masculinised Behaviour of XY Females in a Mammal with Naturally Occuring Sex Reversal. *Sci. Rep.* **6**, 22881; doi: 10.1038/srep22881 (2016).

## Supplementary Material

Supplementary Information

## Figures and Tables

**Table 1:** Number of mice involved in each behavioural test.

Sex	Female			Male
Genotype	XX	XX*	X*Y	XY
Y Maze	12	11	12	13
Resident-intruder paradigm	12	12	12	12
Light-dark Box	12	12	11	12
Open-field	12	12	11	12

**Table 2:** Results of the Two-way choice test.

Total time spent bymales in	XX vs. XX*		XX vs. X*Y		XX* vs. X*Y	
Secondary branches	204.55 +/−73.92	177.16 +/−69.13	167.62 +/−66.76	170.6 +/−81.70	191.92 +/−91.84	163.89 +/−96.95
	V = 30, p = 0.90		V = 47, p = 0.95		V = 38, p = 0.70	
Contact	82.61 +/−36.05	92.5 +/−51.04	80.34 +/−47.18	72.41 +/−50.52	95.23 +/−54.89	68.60 +/−53.90
	V = 26, p = 0.58		V = 58, p = 0.41		V = 47, p = 0.24	
Interaction	44.54 +/−26.83	61.45 +/−31.47	44.14 +/−35.15	42.51 +/−32.65	63.98 +/−47.58	37.21 +/−33.91
	V = 20, p = 0.28		V = 51, p = 0.74		V = 56, p = 0.041	

Total time spent (sec, mean +/−s.e.m.) by males in each side of the apparatus (secondary branches), in contact with the perforated doors, and in interaction with the female through the holes of the door. Statistics: Wilcoxon test. P-values are shown before Bonferroni correction.
