# Corrigendum: Masculinised Behaviour of XY Females in a Mammal with Naturally Occurring Sex Reversal

**DOI:** 10.1038/srep24425

**Published:** 2016-04-22

**Authors:** Paul A. Saunders, Thomas Franco, Camille Sottas, Tangui Maurice, Guila Ganem, Frédéric Veyrunes

Scientific Reports 6: Article number: 2288110.1038/srep22881; published online: 03112016; updated: 04222016

The original version of this Article contained an error in the title of the paper, where the word “Occurring” was incorrectly given as “Occuring”.

In addition, the Acknowledgements section was incomplete.

“We are grateful to J. Britton-Davidian, T. J. Robinson, and J. Watson for their contributions in collecting the founder animals, J. Perez and M. Perriat-Sanguinet for their help in maintaining the breeding colony, P.-A. Crochet for advice on statistical analyses and C.M.-S. Dufour and Y. Latour for helpful discussions. This is a publication ISEM no. 2016-45.”

now reads:

“We are grateful to J. Britton-Davidian, T. J. Robinson, and J. Watson for their contributions in collecting the founder animals, J. Perez and M. Perriat-Sanguinet for their help in maintaining the breeding colony, P.-A. Crochet for advice on statistical analyses and C.M.-S. Dufour and Y. Latour for helpful discussions. This work was supported by the French National Research Agency (ANR grant “SEXYMUS”, No. 10-JCJC-1605), and the Del Duca Foundation (Institut de France, “Subvention scientifique” grant). This is a publication ISEM no. 2016-45.”

These errors have now been corrected in the PDF and HTML versions of the Article.

