# Supplementary material for: Masculinised Behaviour of XY Females in a Mammal with Naturally Occuring Sex Reversal
Source: Sci Rep. 2016 Mar 11;6:22881. doi: 10.1038/srep22881 (PMC4786791; doi:10.1038/srep22881)
Supplement: Supplementary Information [file srep22881-s1.pdf]

# Masculinised Behaviour of XY Females in a Mammal with Naturally Occuring Sex Reversal

**Paul A. Saunders[1,*], Thomas Franco[1], Camille Sottas[1], Tangui Maurice[2], Guila Ganem[1], Frédéric Veyrunes[1]**

[1]Institut des Sciences de l'Evolution de Montpellier, Université de Montpellier, CNRS UMR 5554, IRD, EPHE, France

[2]INSERM U1198, Université de Montpellier, Inserm, EPHE, France

# Supplementary file 1

## RESIDENT-INTRUDER TEST

The effect of genotype on latency to first attack and number of aggressions was analysed using generalised mixed models (*glmer* function in R), with respectively geometric and exponential distribution. *Male ID* was set as a random effect, and *female : male mass ratio* (♀/♂ *mass ratio*) and *male trial number* (one to three) were set as fix covariates. Model simplification was made using Likelihood ratio tests (LRT). Post-hoc comparisons were made using Tukey's HSD tests. Variables with significant effect are highlighted in bold.

### LATENCY TO FIRST ATTACK (SEC)

| XX (mean+/-SD) | XX* | X*Y |
|---|---|---|
| 192.04+/-172.14 | 119.09+/-93.41 | 68.9+/-47.56 |

| Model (fixed effects): ~ Genotype ∗ Mass-ratio + Trial | Variable tested | Statistic |
|---|---|---|
| ~ Genotype + Mass-ratio + Trial | Genotype : ♀/♂ Mass-ratio interaction | $X^2_2$=0.81, p=0.67 |
| ~ Genotype + Trial | ♀/♂ Mass ratio | $X^2_1$=0.2, p=0.66 |
| ~ Genotype | ♂ trial number | $X^2_1$=1.75, p=0.19 |
| ~ 1 | **Genotype** | **$X^2_2$=7.0, p=0.029** |

### NUMBER OF AGGRESSIONS (ATTACKS AND CHASES)

| XX (mean+/-SD) | XX* | X*Y |
|---|---|---|
| 4.36+/-8.07 | 4.33+/-4.96 | 13.63+/-13.03 |

| Model (fixed effects): ~ Genotype ∗ Mass-ratio + Trial | Variable tested | Statistic |
|---|---|---|
| ~ Genotype + Mass-ratio + Trial | Genotype : ♀/♂ Mass-ratio interaction | $X^2_2$=1.67, p=0.43 |
| ~ Genotype + Trial | ♀/♂ Mass ratio | $X^2_1$=0.02, p=0.90 |
| ~ Genotype | ♂ trial number | $X^2_1$=0.02, p=0.89 |
| ~ 1 | **Genotype** | **$X^2_2$=11.00, p=0.004** |

| | **Tukey's HSD tests** | | |
|---|---|---|---|
| | **XX vs. XX*** | **XX vs. X*Y** | **XX* vs. X*Y** |
| Latency to first attack | p=0.45 | p=0.27 | **p=0.037** |
| Number of aggressions | p=0.93 | **p=0.01** | **p=0.03** |

## LIGHT-DARK BOX AND OPEN-FIELD

The effect of sex chromosomes (X* and Y) on variables measured in the light-dark box and the open-field was assessed using independent analyses based on univariate ANOVAS (time spent in light box/central zone and distance covered) or generalised linear models with an exponential distribution (latency before movement). Three covariates were used: the *age* of individuals at the time of the trial; the *group* in which they were tested (from 1 to 12, animals were tested four by four on the same day); the *location* of the individual (upper/lower –right/left). Statistical inference was made using LRT. Significant values (p-value<0.05) are highlighted in bold.

## LIGHT-DARK BOX

### TIME SPENT IN THE LIGHT BOX (SEC)

| XX (mean+/-SD) | XX* | X*Y | XY |
|---|---|---|---|
| 117.66+/-53.79 | 87.80+/-61.09 | 115.02+/-58.56 | 105.41+/-69.96 |

| Model: ~ Y ∗ X* + Group + Age + Position | Variable tested | Statistic |
|---|---|---|
| ~ Y + X* + Group + Age + Position | Y : X* interaction | $F_{1,36}$=2.61 p=0.11 |
| ~ Y + X* + Group + Position | Age | $F_{1,37}$=1e-4, p=0.99 |
| ~ X* + Group + Position | Y chromosome | $F_{1,38}$=1e-3, p=0.97 |
| ~ Group + Position | X* chromosome | $F_{1,39}$=0.11, p=0.89 |
| ~ Position | Group | $F_{1,40}$=0.50, p=0.48 |
| ~ 1 | Position | $F_{3,41}$=1.31, p=0.28 |

### LATENCY BEFORE MOVEMENT (SEC)

| XX (mean+/-SD) | XX* | X*Y | XY |
|---|---|---|---|
| 22.23+/-6.23 | 37.43+/-8.99 | 16.62+/-5.40 | 5.42+/-3.28 |

| Model: ~ Y ∗ X* + Group + Age + Position | Variable tested | Statistic |
|---|---|---|
| ~ Y * X* + Group + Age | Position | $X^2_3$=1.63, p=0.65 |
| ~ Y + X* + Group + Age | Y : X* interaction | $X^2_1$=0.21, p=0.65 |
| ~ Y + X* + Group | Age | $X^2_1$=0.74, p=0.39 |
| ~ Y + Group | X* | $X^2_1$=1.90, p=0.17 |
| ~ Y | Group | $X^2_1$=1.17, p=0.28 |
| ~ 1 | **Y** | **$X^2_1$=6.32, p=0.01** |

### DISTANCE COVERED (CENTIMETRES)

One female removed from dataset (X*Y, distance over 9000 cm)

| XX (mean+/-SD) | XX* | X*Y | XY |
|---|---|---|---|
| 2376.29+/-751.39 | 2380.67+/-976.63 | 2958.56+/-1172.93 | 2871.01+/-975.09 |

| Model: ~ Y ∗ X* + Group + Age + Position | Variable tested | Statistic |
|---|---|---|
| ~ Y + X* + Group + Age + Position | Y : X* interaction | $F_{1,36}$=3.01 p=0.09 |
| ~ Y + X* + Group + Age | Position | $F_{3,37}$=0.14, p=0.93 |
| ~ Y + X* + Age | Group | $F_{1,40}$=2.85, p=0.10 |
| ~ Y + Age | X* | $F_{1,41}$=0.073, p=0.79 |
| ~ Age | **Y** | **$F_{1,43}$=4.64, p=0.04** |
| ~ Y | **Age** | **$F_{1,43}$=3.89, p=0.054** |

# OPEN-FIELD

## TIME SPENT IN THE CENTRAL ZONE (SEC)

| XX (mean+/-SD) | XX* | X*Y | XY |
|---|---|---|---|
| 46.83+/-32.34 | 44.78+/-24.28 | 35.73+/-20.69 | 41.31+/-28.98 |

| Model: ~ Y ∗ X* + Group + Age + Position | Variable tested | Statistic |
|---|---|---|
| ~ Y + X* + Group + Age + Position | Y : X* interaction | $F_{1,37}$=0.08 p=0.77 |
| ~ Y + X* + Age + Position | Group | $F_{1,38}$=0, p=0.99 |
| ~ Y + X* + Age | Position | $F_{3,39}$=0.37, p=0.77 |
| ~ Y + Age | X* | $F_{1,42}$=0.10, p=0.75 |
| ~ Y | Age | $F_{1,43}$=0.47, p=0.50 |
| ~ 1 | Y | $F_{0,44}$=0.80, p=0.38 |

## LATENCY BEFORE MOVEMENT (SEC)

| XX (mean+/-SD) | XX* | X*Y | XY |
|---|---|---|---|
| 6.83+/-4.24 | 10.07+/-4.37 | 18.15+/-10.73 | 11.41+/-5.26 |

| Model: ~ Y ∗ X* + Group + Age + Position | Variable tested | Statistic |
|---|---|---|
| ~ Y + X* + Group + Age + Position | Y : X* interaction | $X^2_1$=0.04, p=0.85 |
| ~ X* + Group + Age + Position | Y | $X^2_1$=0.16, p=0.69 |
| ~ X* + Group +Age | Position | $X^2_3$=3.45, p=0.33 |
| ~ Group + Age | X* | $X^2_1$=0.86, p=0.35 |
| ~ Group | Age | $X^2_1$=0.89, p=0.34 |
| ~ 1 | Group | $X^2_1$=2.92, p=0.09 |

## DISTANCE COVERED (CENTIMETRES)

| XX (mean+/-SD) | XX* | X*Y | XY |
|---|---|---|---|
| 3414.11+/-1559.47 | 3590.74+/-810.42 | 4104.25+/-2165.13 | 4131.48+/-1099.66 |

| Model: ~ Y ∗ X* + Group + Age + Position | Variable tested | Statistic |
|---|---|---|
| ~ Y + X* + Group + Age + Position | Y : X* interaction | $F_{1,37}$=0.53 p=0.47 |
| ~ Y + Group + Age + Position | X* | $F_{1,38}$=7e-4, p=0.98 |
| ~ Y + Group + Position | Age | $F_{1,39}$=0.08, p=0.78 |
| ~ Y + Group | Position | $F_{3,40}$=1.13, p=0.35 |
| ~ Y | Group | $F_{1,43}$=2.39, p=0.13 |
| ~ 1 | Y | $F_{1,44}$=2.14, p=0.15 |
